# Long‐term outcomes of relapsed/refractory double‐hit lymphoma (r/r DHL) treated with CD19/22 CAR T‐cell cocktail therapy

**DOI:** 10.1002/ctm2.176

**Published:** 2020-09-17

**Authors:** Jia Wei, Zekai Mao, Na Wang, Lifang Huang, Yang Cao, Weimin Sun, Xiaolu Long, Jiaqi Tan, Chunrui Li, Yi Xiao, Chaojiang Gu, Shangkun Zhang, Yicheng Zhang, Tongcun Zhang, Jianfeng Zhou, Liang Huang

**Affiliations:** ^1^ Department of Hematology, Tongji Hospital, Tongji Medical College Huazhong University of Science and Technology Wuhan Hubei China; ^2^ Immunotherapy Research Center for Hematologic Diseases of Hubei Province Wuhan Hubei China; ^3^ College of Life Science and Health Wuhan University of Science and Technology Wuhan Hubei China; ^4^ Wuhan Bio‐Raid Biotechnology Co., Ltd Wuhan Hubei China

Dear Editor,

Previously, we reported the safety and efficacy of sequential infusion of CD19/22 chimeric antigen receptor (CAR) T‐cells immunotherapy in treatment of relapsed/refractory (r/r) B‐cell lymphoma at first time and suggested its potential role in certain subsets of patients who carried high‐risk genetic or chromosome aberrations.^1^ However, the long‐term outcomes of r/r double‐hit lymphoma (DHL) being treated by this immunotherapy has not been fully elucidated. DHL with *c‐MYC* and *BCL2* or *BCL6* gene rearrangements have been defined as a new separate subtype in 2016 revised WHO classification of lymphoid neoplasms.^2^ Currently, no consensus has been reached in the treatment of DHL. Patients have inferior outcomes treated with conventional or targeted chemotherapy^3^ and no significant benefit can be obtained from further consolidative transplantation.^4^ If DHL patients have either refractory or relapsed diseases after initial treatments, the prognosis will not be improved by noncross‐resistant second‐line chemotherapy and subsequent autologous‐stem cell transplantation (ASCT).^5^ One investigation reported 50% response rate in 19 primary refractory and/or relapsed double‐hit lymphoma (r/r DHL) patients treated with tisagenlecleucel.^6^ In ZUMA‐1 trial, seven patients with confirmed high‐grade B‐cell Lymphoma (HGBL) or DHL had 90% objective responses (OR) treated with axicabtagene ciloleucel.^7^ Long‐term survival data, however, were not reported in these studies. Recently, one DHL case was reported to maintain complete response (CR) for more than 1 year by treating with axicabtagene ciloleucel, indicating the potential survival benefit with CAR T‐cell immunotherapy.^8^ Although ASCT still plays a potentially curative role in treatment of most types of r/r B‐cell lymphoma, treatment of r/r DHL patients who hadworse prognosis as a higher risk subtype need incorporate with more investigational strategies rather than standard ASCT alone.^9^ Whether ASCT following with CAR T‐cell infusion can improve the prognosis of r/r DHL patients remains unclear.

From February 2017 through May 2019, 12 consecutive r/r DHL patients (Table 1) were enrolled into two separated ongoing clinical trials (Figure 1A): CD19/22 CAR T‐cell immunotherapy (Trial A, eight patients) and the infusion of CD19/22 CAR T‐cell immunotherapy following ASCT (Trial B, six patients), including two patients switching from Trial A. The baseline characteristics of enrolled patients across these two trials were well balanced (Table S1). The overall objective response rate (ORR) at month 3 post CAR T‐cell immunotherapy was 83.3% (10/12, 95% CI: 55.2‐97.0%), including three cases of CR (25%) and seven cases of partial response (PR) (58.3%). The best ORR was 83.3% (10/12, 95% CI: 55.2‐97.0%) with 50% best CR rate and 33.3% best PR rate. Comparable responses were achieved across different subtype‐specific impact (Table S2). Among the patients in Trial A, 75.0% patients (6/8, 95%CI: 40.9‐95.6%) achieved OR at month 3 post CAR T‐cell immunotherapy, including one with CR. Among five with PR, two had PD at 3.1 and 8.7 months, respectively. In Trial B, the ORR at month 3 post CAR T‐cell immunotherapy was 100% (95%CI: 61‐100%), including one with CR. Among those five with PR in Trial A, two patients were enrolled in Trial B when early B‐cell recovery had been detected. Only one patient who had achieved CR in Trial B had PD at 5.9 months post CAR T‐cells infusion and eventually died at 12.2 months. Figure 1B presents duration of response and long‐term outcomes of all enrolled patients.

**TABLE 1:** r/r DHL patients receiving sequential infusion of CAR19/22 T‐cell cocktail therapy (Trial A and Trial B)

No.	Age (Y)	Gender	Trial	Date of infusion	Conditioning regimen*	Dosage CD19 CAR‐T	Dosage CD22 CAR‐T	CRS grade^†^	CRES grade^‡^	Best response#	Second CAR T with ASCT
1^§^	36	F	Trial A	2017/02/25	FC	4.0 × 10^6^/kg	4.0 × 10^6^/kg	2	1	PR	Yes
			Trial B	2017/08/25	BEAM	4.0 × 10^6^/kg	4.0 × 10^6^/kg	2	0	CR	
2^§^	51	M	Trial A	2017/11/18	FC	3.7 × 10^6^/kg	5.2 × 10^6^/kg	3	0	PR	Yes
			Trial B	2018/04/12	BEAM	3.7 × 10^6^/kg	5.2 × 10^6^/kg	0	1	CR	
3	43	F	Trial A	2018/01/21	FC	7.5 × 10^6^/kg	2.5 × 10^6^/kg	1	0	CR	No
4	47	F	Trial A	2018/01/27	FC	4.0 × 10^6^/kg	8.0 × 10^6^/kg	1	1	PR	No
5	33	M	Trial A	2017/12/15	FC	5.0 × 10^6^/kg	5.2 × 10^6^/kg	3	0	PD	No
6	45	M	Trial A	2018/03/30	FC	6.8 × 10^6^/kg	4.3 × 10^6^/kg	4	0	PD	No
7	46	M	Trial A	2018/11/07	FC	3.3 × 10^6^/kg	3.5 × 10^6^/kg	1	1	PR	No
8	67	M	Trial A	2019/05/16	FC	3.4 × 10^6^/kg	3.7 × 10^6^/kg	0	0	PR	No
9	41	F	Trial B	2019/05/20	BEAM	2.8 × 10^6^/kg	2.6 × 10^6^/kg	1	0	PR	No
10	39	F	Trial B	2019/03/31	BEAM	4.9 × 10^6^/kg	4.9 × 10^6^/kg	1	0	PR	No
11	24	F	Trial B	2018/11/25	BEAM	4.0 × 10^6^/kg	4.0 × 10^6^/kg	0	0	CR	No
12	41	M	Trial B	2017/09/02	BEAM	3.6 × 10^6^/kg	4.3 × 10^6^/kg	1	0	CR	No

Y, Years; F, female; M, male; CRS, cytokine release syndrome; CRES, CAR T‐cell‐related encephalopathy syndrome; FC, fludarabine and cyclophosphamide; BEAM, bis‐carmusitine, etoposide, cytarabine and melphalan; CR, complete response; PD, progressive disease; PR, partial response.

^§^ Two patients who had achieved a PR after CAR T‐cell cocktail infusion received second‐round infusion following ASCT when early B‐cell recovery was detected. They continued to have an ongoing CR after 8.7 and 9.6 months, respectively.

* Conditioning regimen: In CAR19/22 T‐cell cocktail therapy (Trial A), patients were given fludarabine (25 mg/m^2^) and cyclophosphamide (300 mg/m^2^) (FC regimen) for 3 days (days −4 to −2) as lymphodepletion chemotherapy. CAR19 and CAR22 T cells were infused separately on successive days from day zero as reported previously. In CAR19/22 T‐cell cocktail infusion following ASCT (Trial B), patients were given standard dose of BEAM regimen (300 mg/m^2^ of bis‐carmusitine, −6 day; 200 mg/m^2^ of etoposide −5 to −2 days; 400 mg/m^2^ of cytarabine −5 to −2 days, and 140 mg/m^2^ of melphalan −1 day) as myeloablative chemotherapy. CAR19 and CAR22 T cells were infused 2∼6 days (days +2 to +6) after autologous stem cell infusion (day 0).

^†^ Cytokine release syndrome (CRS) was graded as the scale proposed by ASTCT Consensus Grading for Cytokine Release Syndrome and Neurologic Toxicity Associated with Immune Effector Cells.

^‡^ CAR T‐cell‐related encephalopathy syndrome (CRES) was evaluated according to the National Cancer Institute Common Terminology Criteria for Adverse Events V4.03

^#^ Response assessments were defined according to the National Comprehensive Cancer Network guidelines and Lugano Treatment Response Criteria.

**FIGURE 1:**
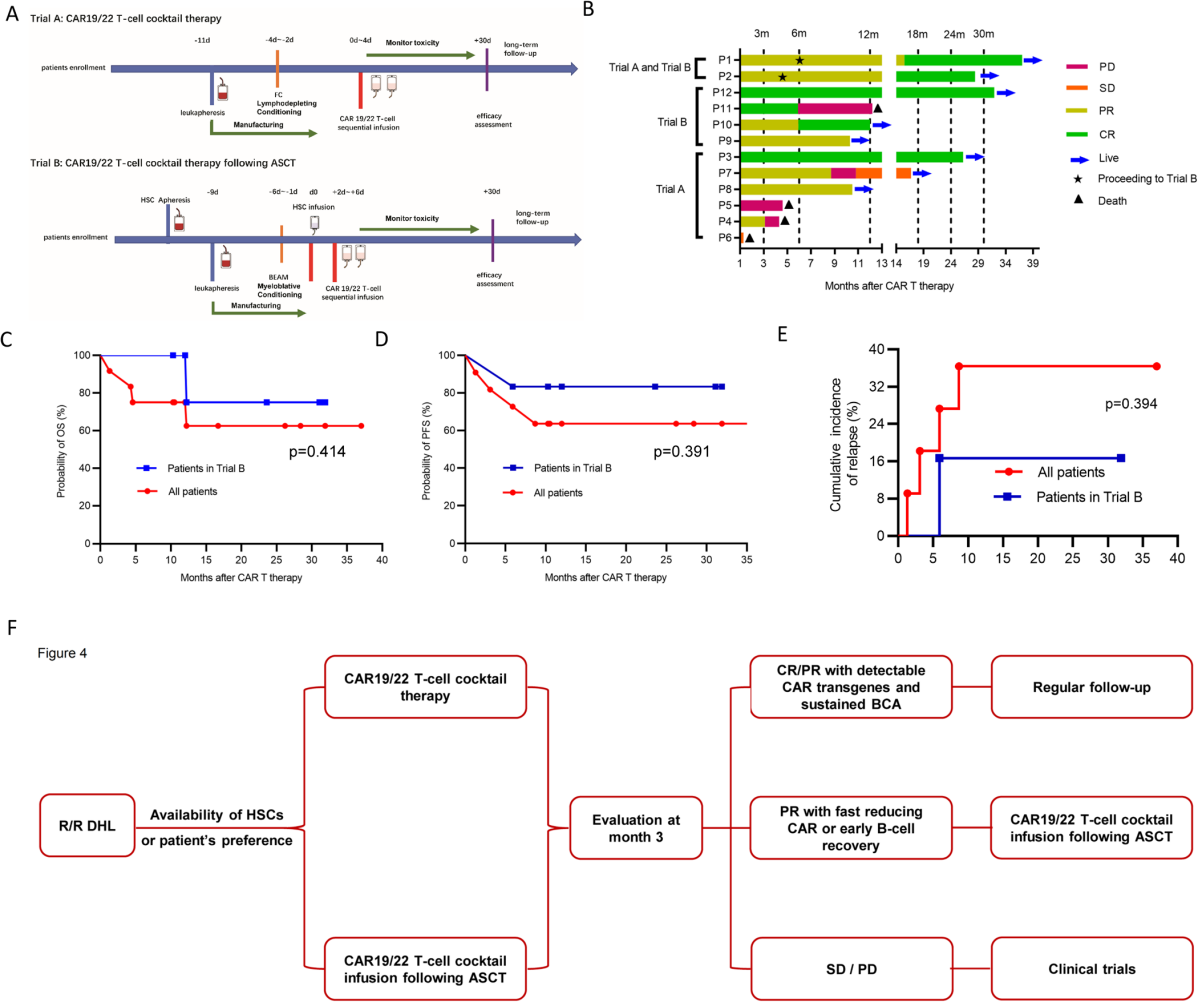
A, Schematic diagram of study procedures. After providing written informed consent, all eligible patients with relapsed/refractory double‐hit lymphoma underwent leukapheresis. If needed, bridging therapy was allowed. In CD19/22 CAR T‐cell immunotherapy (Trial A), patients were given 25 mg/m^2^ of fludarabine and 300 mg/m^2^ cyclophosphamide (FC regimen) for consecutive 3 days (−4 days to −2 days) as lymphodepletion chemotherapy. CD19/22 CAR T‐cells were infused separately on the following successive days starting from d0. In CD19/22 CAR T‐cell infusion following ASCT (Trial B), patients were given standard dose of BEAM regimen (300 mg/m^2^ of bis‐carmusitine, −6 day; 200 mg/m^2^ of etoposide −5 to −2 days; 400 mg/m^2^ of cytarabine −5 to −2 days, and 140 mg/m^2^ of melphalan −1 day) as myeloablative conditioning regimen. CD19/22 CAR T cells were infused 2‐6 days (days +2 to +6) after autologous stem cells infusion (d0). B, Duration of response and outcomes of all enrolled patients. The cutoff date (the last day of follow‐up) was March 31, 2020. PD represents progressive disease; SD represents stable disease; PR represents partial response; CR represents complete response. C, The Kaplan‐Meier curve for overall survival (OS) of all enrolled patients (red) and of patients in Trial B (blue); tick marks represent censored data. D, The Kaplan‐Meier curve for progression‐free survival (PFS) of all enrolled patients. PFS of all enrolled patients (red) and of patients in Trial B (blue); tick marks represent censored data. E, Cumulative incidence curve of relapse of enrolled patients. Cumulative incidence of relapse of all enrolled patients (red) and of patients in Trial B (blue). F, A proposed novel strategy for the treatment of r/r DHL with CAR T‐cell immunotherapy. According to the availability of HSCs and the preference of patients, patients with r/r DHL are treated with CAR19/22 T‐cell cocktail alone or following ASCT. After treatment, treatment responses, lentiviral copies of CAR transgenes, and B‐cell kinetics are regularly evaluated. Active follow‐up is reasonable for patients with detectable CAR T cells and sustained B‐cell aplasia. For patients in partial response (PR) but with fast reducing CAR T cells or with early B‐cell recovery, response‐guided second‐round CAR T‐cell infusion following ASCT could be applied. For patients in stable or progressive disease (SD/PD), other clinical trials are highly recommended.

In the present study, promising results in r/r DHL treatet with CAR T‐cell therapy have been achieved with low‐grade (grade ≤2) cytokine release syndrome (CRS) and CAR T‐cell‐related encephalopathy syndrome (CRES), respectively (Table 1 and Table S3). The median follow‐up time was 12.1 months (range, 1.3‐37.0) and both median overall survival (OS) and progression‐free survival (PFS) were not reached in this cohort (Figure 1C and D). The 1‐year OS rate was 75.0% (95% CI: 40.8‐91.2) for all enrolled patients. However, the median follow‐up time of patients in Trial B was 17.9 months (range, 10.3‐31.9) and the estimated 2‐year OS rate was 75.0% (95% CI: 12.8‐96.1) (Figure 1C). The 1‐year PFS rate was 63.6% (95% CI: 29.7‐84.5) among all enrolled patients; however, for patients in Trial B, the estimated 2‐year PFS rate was 83.3% (95% CI: 27.3‐97.5, Figure 1D). The estimated 1‐year incidence of relapse rate for all patients was 36.4% (95% CI: 15.5‐70.3, Figure 1E). Only one patient had disease relapse in patients in Trial B until the cutoff date. Particularly, when compared with patients received CAR19/22 T‐cell cocktail alone, there were trends toward numerical higher response rate and survival time, fewer relapses and adverse effects, longer persistence of CAR19 and CAR22 transgenes, and B‐cell aplasia (BCA) (Figure S1 and Table S4) in patients who undergone CAR19/22 T‐cell cocktail following ASCT. Further testing in larger patient cohorts with different ethnicity is required.

SCHOLAR‐1, the largest pooled retrospective investigation on r/r diffuse large B‐cell lymphoma (DLBCL),^10^ indicated that patients who obtained CR status after salvage chemotherapy had superior OS than nonresponders. Remarkably extended PFS and OS of patients in CR status was achieved at month 3 post CAR T‐cell immunotherapy when compared to patients in PR status.^1^ Therefore, to achieve a CR after second‐ or late‐line salvage therapy is the principal goal for r/r DLBCL patients. In our study, 66.7% DHL patients achieved a PR at month 3 post CD19/22 CAR T‐cell infusion. Although patients who achieved a PR after CAR T‐cell infusion could continue to have a CR without additional treatment, two patients with PR in Trial A received second‐round CAR19/22 T‐cell cocktail infusion following ASCT when early B‐cell recovery had been detected. Both patients thereafter achieved an ongoing CR at 8.7 and 9.6 months, respectively, indicating response‐guided second‐round CAR T‐cell infusion following ASCT could reinforce the efficiency of first‐round CAR T‐cell infusion. Hence, response assessment and kinetics screening for CAR transgenes and B‐cell recovery are critical for patients after CAR T‐cell infusion. Regular follow‐up is proper for patients with detectable CAR T‐cells and sustained BCA, but response‐guided second‐round CAR T‐cell infusion following ASCT is a reasonable strategy for patients in PR but with fast reducing CAR T‐cells or with early B‐cell recovery (Figure 1F). This proposed strategy could also be extended to the treatment for high‐risk B‐NHL and should be investigated in future clinical trials.

In conclusion, this is the first dedicated and largest case series looking at r/r DHL patients who were treated with CD19/22 sequential CAR T‐cells infusion. Our data suggested that CD19/22 CAR T‐cell immunotherapy alone or following ASCT can improve the long‐term outcome of r/r DHL. It provides a promising novel therapeutically option for r/r DHL.

## CONFLICT OF INTERESTS

The authors declare that there is no conflict of interest.

## ETHICS APPROVAL AND CONSENT TO PARTICIPATE

This study was approved by the Medical Ethics Committee of Tongji Hospital, Tongji Medical College, Huazhong University of Science and Technology (TJ‐IRB20160310). All patients gave their written informed consents in accordance with the Declaration of Helsinki. This study is registered at www.chictr.org.cn as ChiCTR‐OPN‐16008526 and ChiCTR‐OPN‐ 16009847. The authors have obtained consent to publish from the participant to report individual patient data.

## FUNDING INFORMATION

Key Program of the National Natural Science Foundation of China; Grant Number: 81830008; National Natural Science Foundation of China; Grant Numbers: 81670152, 81873427, and 81600120).

## AUTHOR CONTRIBUTIONS

Jianfeng Zhou and Liang Huang designed and supervised the clinical study; Tongcun Zhang, Chaojiang Gu, and Shangkun Zhang supervised the CAR T‐cell production; Na Wang and Yang Cao conducted preclinical validation and quality control; Jia Wei, Zekai Mao, Weimin Sun, Xiaolu Long, Jiaqi Tan, and Lifang Huang collected clinical data; Jia Wei, Zekai Mao, and Liang Huang analyzed data, wrote, and revised the manuscript; Jia Wei and Zekai Mao performed statistical analyses; Jianfeng Zhou, Liang Huang, Yang Cao, Chunrui Li, Yi Xiao, Yicheng Zhang, Jia Wei, Na Wang, Zekai Mao, and Weimin Sun enrolled and took care of the patients.

## Supporting information

Supporting Information

## Data Availability

The data that support the findings of this study are available in the Supporting Information. All published data and material are available upon request from the corresponding authors.
