# Supplementary material for: Long‐term outcomes of relapsed/refractory double‐hit lymphoma (r/r DHL) treated with CD19/22 CAR T‐cell cocktail therapy
Source: Clin Transl Med. 2020 Sep 17;10(5):e176. doi: 10.1002/ctm2.176 (PMC7507504; doi:10.1002/ctm2.176)
Supplement: Supplementary file 1 — Supporting Information [file CTM2-10-e176-s001.docx]

Supplement to ***Jia Wei, et al*** ***Long-term outcomes of relapsed/refractory double-hit lymphoma (r/r DHL) treated with CD19/22 CAR T-cell cocktail therapy.***

This Supplemental Materials has been provided by the authors to give readers additional information about their work.

**Supplemental Materials**

**Contents:**

SUPPLEMENTAL Methods 3

SUPPLEMENTAL Results 5

[Supplemental tables 6](#_Toc33305267)

[Table S1. Balanced characteristics of the enrolled patients in each trial 6](#_Toc33305268)

[Table S2. Comparable responses achieved across different subgroups 7](#_Toc33305269)

[Table S3. Comparison of CRS Grade between Trial A and Trial B 8](#_Toc33305270)

[Table S4. Persistence times of CAR transgenes and B cell aplasia 9](#_Toc33305270)

[Supplemental Figures 10](#_Toc33305271)

[Figure S1. Persistence times of CAR transgenes and B-cell aplasia 10](#_Toc33305273)

**Supplemental Methods**

***Study design and patients***

Due to the preference of patients and the availability of hematopoietic stem cells (HSCs), patients with r/r DHL were enrolled in trials with CD19/22 CAR T-cell cocktail alone (Trial A) or following ASCT (Trial B), 2 independent ongoing clinical trials, respectively. These 2 trials were approved by the institutional review board of Tongji Hospital, Tongji Medical College, Huazhong University of Science and Technology, and registered with the Chinese Clinical Trial Registry (ChiCTR, number ChiCTR-OPN-16008526 and 16009847). The diagnosis of DHL was confirmed with FISH. Before enrollment, dual expression of CD19 and CD22 on malignant B cells was re-assessed by flow cytometry or immunohistochemistry (IHC). Good performance status (ECOG-PS ≤ 2), essentially normal organ function, measurable disease and a life expectancy of 12 weeks or more were necessary for eligibility, while patients with uncontrollable infection, active graft-versus-host disease (GVHD) or clinically evident neurological lesions were excluded. Written informed consent was obtained from each participant, in compliance with the Declaration of Helsinki.

***Lentiviral construction and in vitro validation***

As previous reported, the third-generation CAR utilized in this trial was composed of a single chain variable fragment derived from a murine monoclonal antibody against human CD19 or CD22, two costimulatory domains from CD28 and 4-1BB, and CD3ζ chain as activation domain. Validation of the CAR constructs and procedures for cell production and quality-control assays are described previously.

***Conditioning regimens, sequential infusion of CAR22 and CAR19 T-cell***

In CD19/22 CAR T-cell therapy (Trial A), patients were given fludarabine (25mg/m^2^) and cyclophosphamide (300mg/m^2^) (FC regimen) for 3 days (days -4 to -2) as lymphodepletion chemotherapy. CAR19 and CAR22 T-cells were infused separately on successive days from day zero as reported previously. In CD19/22 CAR T-cell infusion following ASCT (Trial B), patients were given standard dose of BEAM regimen (300mg/m^2^ of bis-carmusitine, -6 day; 200mg/m^2^ of etoposide -5 to -2 days; 400mg/ m^2^ of cytarabine -5 to -2 days, and 140 mg/m^2^ of melphalan -1 day) as myeloablative chemotherapy. CAR19 and CAR22 T-cells were infused 2~6 days (days +2 to +6) after autologous stem cell infusion (day 0).

***Safety evaluation and follow-up***

*In vivo* expansion of CAR19 and CAR22 T-cells were measured by droplet digital polymerase chain reaction (ddPCR). Cytokines were assessed according to the manufacturer’s instruction. Cytokine release syndrome (CRS) was graded as the scale proposed by ASTCT Consensus Grading for Cytokine Release Syndrome and Neurologic Toxicity Associated with Immune Effector Cells. CAR T-cell-related encephalopathy syndrome (CRES) and other adverse events (AEs) were evaluated according to the National Cancer Institute Common Terminology Criteria for Adverse Events V4.03. Staging and response assessments were defined according to the National Comprehensive Cancer Network guidelines and Lugano Treatment Response Criteria. All patients were followed up until they died, lost to follow-up, or withdrew consent. Disease assessments were based on either computed tomography (CT) or PET/CT at baseline (pre-HSCT), 30 days, and every three months after initial CAR T-cell infusion. Circulating tumor DNA (ctDNA) monitoring for disease assessment was optional. Additional imaging studies were performed when clinically indicated.

***Statistical analysis***

All data were analyzed using SPSS 22.0 software (SPSS Inc., Chicago, IL, USA). PFS was defined as the time from first infusion to disease progression or death. Both PFS and OS were censored at last follow-up. The survival rate was analyzed using the Kaplan-Meier method. Differences in OS and PFS between different groups were estimated by the log-rank test. Differences between groups were performed by chi-square statistic or Fisher’s exact test for categorical variables, and by independent sample t test for continuous variables. *P* values less than 0.05 (two-tailed) were statistically significant.

**Supplemental Results**

***Adverse effects***

Most patients (78.5%) have transient and mild to moderate CRS in both trials. The median onset of CRS occurred on day 3 (range:2~7), with median resolution on day 12 (range: 7~21) post-infusion. Although there is no difference of CRS incidence between these 2 trials, all severe (grade≥3) cases (21.4%) occurred in trial A (Table S3). We used tocilizumab, corticosteroid and/or plasma exchange for toxicity management. One patient in Trial A developed grade 4 CRS 5 days post-infusion and then complicated with septic shock (cytomegalovirus and invasive fungus infection). He eventually died of multiple organ dysfunction syndrome 43 days post-infusion. No patients developed severe CRES (grade ≥ 3) after CAR T-cells infusion. A total of 4 patients in both trials had grade 1 CRES which resolved spontaneously. We also evaluated infection in CAR T-cell therapy. Cytomegalovirus infection was the most common virus infection during treatment (21.4%) and could be controlled by intravenous infusion of ganciclovir. No patients died within 30 days due to any reasons.

***Persistence of functional CAR T-cells***

Among all enrolled patients in both trials, the median persistence times of CAR19 and CAR22 T- cells were 4.6 months (range, 0.2~26.1) and 5.7 months (range, 0.2~16.5), respectively (Table S4). The median persistence times of CAR19 T-cells and CAR22 T-cells in Trial A (2.7 months for CAR19 T-cells and 4.4 months for CAR22 T-cells) were numerically shorter than the times in Trial B (8.4 months for CAR19 T-cells and 8.4 months for CAR22 T-cells). The median time for B cell aplasia (BCA) among all enrolled patients in these 2 trials was 5.4 months (range: 1.3~26.1). The median time of BCA in Trial A was numerically less than those in Trial B (3.8 months vs 9.6 months). However, no significant differences were found between the persistence times of CAR transgenes and B cell aplasia among these 2 trials (*P* > 0.05, Table S4).

**Supplemental tables**

**Table S1. Balanced characteristics of the enrolled patients in each trial**

| **Characteristics** | **All^*^** | **Trail A^*^** | | **Trail B^*^** | ***P*** |
| --- | --- | --- | --- | --- | --- |
| Age, median (range) | 43 (24-67) | 46 (33-67) | | 40 (24-51) | 0.280 |
| Gender (M/F) | 6/6 | 5/3 | | 2/4 |  |
| Disease status |  |  | |  |  |
| Primary refractory | 6 (50.0) | 4 | | 4 | 0.533 |
| First relapse | 2 (16.7) | 0 | | 0 |  |
| Second relapse | 2 (16.7) | 2 | | 2 | 0.186 |
| ≥ Third relapse | 2 (16.7) | 2 | | 0 |  |
| Prior auto transplantation | 1 (8.3) | 1 | | 0 |  |
| Disease stage^‡^ |  |  | |  | 0.347 |
| I or II | 3 (25.0) | 1 | | 2 |  |
| III or IV | 9 (75.0) | 7 | | 4 |  |
| IPI risk score |  |  | |  | 0.334 |
| 0-2 | 4 (33.3) | 2 | | 3 |  |
| 3-4 | 8 (66.7) | 6 | | 3 |  |
| Cell of origin |  |  | |  | 0.124 |
| GCB | 9 (75.0) | 7 | | 3 |  |
| Non-GCB | 3 (25.0) | 1 | | 3 |  |
| Lines of previous therapy | | |  | |  |
| 2 | 5 (41.7) | 2 | | 3 | 0.334 |
| 3 | 3 (25.0) | 3 | | 0 | 0.640 |
| >3 | 4 (33.3) | 3 | | 3 |  |
| Gene mutations^‡^ |  |  | |  |  |
| *c-MYC* | 6 (50.0) | 3 | | 4 | 0.280 |
| *KMT2D* | 6 (50.0) | 4 | | 4 | 0.533 |
| *CREBBP* | 4 (33.3) | 3 | | 3 | 0.640 |
| *CARD11* | 2 (16.7) | 1 | | 1 | 0.584 |
| *TP53* | 2 (16.7) | 1 | | 2 | 0.157 |
| *EZH2* | 2 (16.7) | 1 | | 1 | 0.584 |
| *BCL2* | 1 (8.3) | 0 | | 1 | 0.140 |
| *MYD88* | 1 (8.3) | 1 | | 1 | 0.584 |

**^*^**Values are presented as number (%) unless otherwise indicated; **^†^**According to Ann Arbor staging; ^‡^Determined by next-generation sequencing. DHL, Double-hit lymphoma; M, male; F, female; IPI, international prognostic index; GCB, germinal center B-cell like.

**Table S2. Comparable responses achieved across different subgroups**

| **Characteristics** | **No.** | **Best ORR** | | | | ***P*** |
| --- | --- | --- | --- | --- | --- | --- |
|  |  | **No.** | **ORR (%)** | | **95% CI** |  |
| Total | 12 | 10 | 83 | 0.59-1.08 | |  |
| Gender | | | | | | |
| Female | 6 | 6 | 100 | 1.00-1.00 | | 0.12 |
| Male | 6 | 4 | 67 | 0.13-1.21 | |  |
| Age (years) | | | | | | |
| < 30 | 1 | 1 | 100 | 1.00-1.00 | | 0.64 |
| ≥ 30 | 11 | 9 | 82 | 0.55-1.08 | |  |
| Disease status**^†^** | | | | | | |
| I or II | 3 | 3 | 100 | 1.00-1.00 | | 0.37 |
| III or IV | 9 | 7 | 78 | 0.44-1.12 | |  |
| IPI risk score | | | | | | |
| 0 - 2 | 4 | 4 | 100 | 1.00-1.00 | | 0.27 |
| 3 - 4 | 8 | 6 | 75 | 0.36-1.14 | |  |
| Cell of origin | | | | | | |
| GCB | 9 | 7 | 78 | 0.44-1.12 | | 0.26 |
| non-GCB | 3 | 3 | 100 | 1.00-1.00 | |  |
| Disease status | | | | | | |
| Refractory | 7 | 7 | 100 | 1.00-1.00 | | 0.07 |
| Relapse | 5 | 3 | 60 | 0.08-1.28 | |  |
| Previous radiotherapy | | | | | | |
| With | 2 | 2 | 100 | 1.00-1.00 | | 0.49 |
| Without | 10 | 8 | 80 | 0.50-1.10 | |  |
| Lines of pervious therapy | | | | | | |
| 2 | 5 | 4 | 80 | 0.25-1.36 | | 0.19 |
| ≥ 2 | 7 | 6 | 86 | 0.51-1.21 | |  |

**^†^**According to Ann Arbor staging; No., number; ORR, objective response rate; CI, confidence interval.

**Table S3. Comparison of CRS Grade between Trial A and Trial B**

|  | **Low-grade (0 ~ 2)** | **High-grade (≥ 3)** | ***P*** |
| --- | --- | --- | --- |
| Trial A | 5 | 3 | 0.091 |
| Trial B | 6 | 0 |  |

CRS, cytokine release syndrome.

**Table S4.** **Persistence times of CAR transgenes and B cell aplasia (months)**

| **Persistence times,**  **Median (range)** | **All patients** | **Patients in Trial A** | **Patients in Trial B** | ***P*^††^** |
| --- | --- | --- | --- | --- |
| CAR19^*^ | 4.6 (0.2-26.1) | 2.7 (0.2-14.4) | 8.4 (1.2-25.1) | 0.1583 |
| CAR22^*^ | 5.7 (0.2-16.5) | 4.4 (0.2-11.2) | 8.4 (1.2-16.5) | 0.1648 |
| B cell aplasia^†^ | 5.4 (1.3-26.1) | 3.8 (1.3-12.0) | 9.6 (3.1-26.1) | 0.0827 |

^*^CAR19/22, lentivirus copies of CAR19 and CAR22; the existence of CAR was defined as greater than 50 copies/ug; ^†^B cell aplasia, defined as <1% B cells/ WBCs or < 3% B cells/lymphocytes (the lower limit of normal for blood B-cell levels); ^††^*P*, compared between Trial A and Trial B by independent sample t test.

**Supplemental Figures**


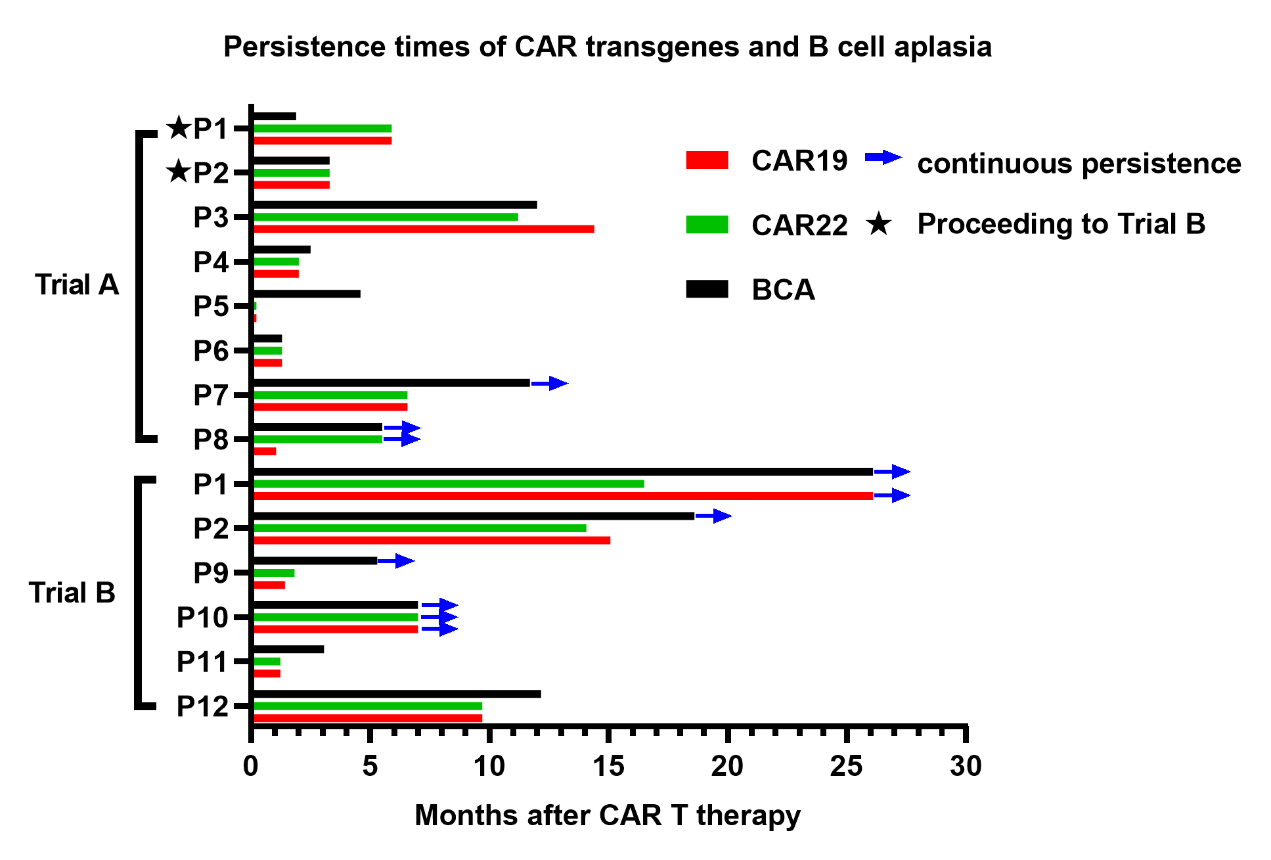


**Figure S1. Persistence times of CAR transgenes and B-cell aplasia.** CAR transgenes were assessed by lentivirus copies of CAR19 and CAR22. The existence of CAR transgenes was defined as greater than 50 copies/ug. † B cell aplasia was defined as <1% B cells/ WBCs or < 3% B cells/lymphocytes (the lower limit of normal for blood B-cell levels). Two patients who had achieved a PR after CAR T-cell cocktail infusion (Trial A) received second-round infusion following ASCT (Trial B) when early B-cell recovery was detected. They continued to have an ongoing CR after 8.7 and 9.6 months, respectively.
